# Extract of *Stellerachamaejasme L*(ESC) inhibits growth and metastasis of human hepatocellular carcinoma via regulating microRNA expression

**DOI:** 10.1186/s12906-018-2123-y

**Published:** 2018-03-20

**Authors:** Xiaoni Liu, Shuang Wang, Jianji Xu, Buxin Kou, Dexi Chen, Yajie Wang, Xiaoxin Zhu

**Affiliations:** 1 Beijing Institute of Hepatology and Beijing YouAn Hospital, Capital Medical University, No 8 Xi TouTiao, You An Men Wai, Feng Tai Qu, Beijing, 100069 China; 2 Institute of Chinese Materia Medica, China Academy of Chinese Medical Sciences, No 16 Nan Xiao Jie, Dong Zhi Men Nei, Dong Cheng Qu, Beijing, 100700 China

**Keywords:** Hepatocellular carcinoma, microRNA, *Stellerachamaejasme L*, Target gene, MCL1, SALL4, BCL2

## Abstract

**Background:**

MicroRNAs(miRNAs)are involved in the initiation and progression of hepatocellular carcinoma. ESC, an extract of *Stellerachamaejasme L*, had been confirmed as a potential anti-tumor extract of Traditional Chinese Medicine. In light of the important role of miRNAs in hepatocellular carcinoma, we questioned whether the inhibitory effects of ESC on hepatocellular carcinoma (HCC) were associated with miRNAs.

**Methods:**

The proliferation inhibition of ESC on HCC cells was measured with MTT assay. The migration inhibition of ESC on HCC cells was measured with transwell assay. The influences of ESC on growth and metastasis inhibition were evaluated with xenograft tumor model of HCC. Protein expressions were measured with western blot and immunofluorescence methods and miRNA profiles were detected with miRNA array. Differential miRNA and target mRNAs were verified with real-time PCR.

**Results:**

The results showed that ESC could inhibit proliferation and epithelial mesenchymal transition (EMT) in HCC cells in vitro and tumor growth and metastasis in xenograft models in vivo. miRNA array results showed that 69 differential miRNAs in total of 429 ones were obtained in MHCC97H cells treated by ESC. hsa-miR-107, hsa-miR-638, hsa-miR-106b-5p were selected to be validated with real-time PCR method in HepG2 and MHCC97H cells. Expressions of hsa-miR-107 and hsa-miR-638 increased obviously in HCC cells treated by ESC. Target genes of three miRNAs were also validated with real-time PCR. Interestingly, only target genes of hsa-miR-107 changed greatly. ESC downregulated the MCL1, SALL4 and BCL2 gene expressions significantly but did not influence the expression of CACNA2D1.

**Conclusion:**

The findings suggested ESC regressed growth and metastasis of human hepatocellular carcinoma via regulating microRNAs expression and their corresponding target genes.

## Background

MicroRNAs (miRNAs) are small non-coding RNAs characterized by a length of 18–25 nucleotides and capable of binding to complementary 3′UTR regions of their target genes, thereby modulating the transcription of the target mRNA [1]. Mounting evidences have demonstrated that miRNAs are involved in the initiation and progression of several types of human cancer, including hepatocellular carcinoma (HCC), which is one of the most common types of cancer and the third leading cause of cancer-related mortality worldwide [2]. It was recently demonstrated that miRNAs played critical roles in HCC progression and directly contributed to tumor cell proliferation, avoidance of apoptotic cell death and metastasis by targeting a large number of specific mRNAs. miRNAs may undergo aberrant regulation during carcinogenesis and act as oncogenes or tumor suppressor genes in HCC [3–6].

ESC, an extract of *StellerachamaejasmeL*, rich in isomers of *Chamaejasminor, neochamaejasmine* and *Sikokianin* [7], had antitumor effects by activating apoptosis pathway and reversed EMT of tumor cells induced by TGF-β via inhibition of Smad signaling pathway in our previous studies [8, 9]. ESC had been confirmed as a potential anti-tumor extract of Traditional Chinese Medicine.

In light of the important role of miRNAs in hepatocellular carcinoma, we questioned whether the inhibitory effects of ESC on hepatocellular carcinoma were associated with miRNAs. To figure out this question, this study was performed to evaluate modulatory effect of ESC on miRNAs expression in hepatocellular carcinoma in order to clarify the molecular mechanisms of ESC on hepatocellular carcinoma.

## Methods

### Preparation of ESC

ESC was provided and identificated by Hong Bin Xiao (China Academy of Chinese Medical Sciences, Beijing, People’s Republic of China), which has been deposited in Institute of Chinese Materia Medica, China Academy of Chinese Medical Sciences(Deposition number:ICMM-001). The extract method was as follows: *Stellerachamaejasme L* herbal medicine was extracted 3 times with ethanol. Meanwhile, the concentrated liquid (volatile to non-alcohol taste) was washed on a polyamide column with 60% ethanol, and then decompressively recycled and vacuum dried at room temperature. The final compound obtained was ESC.

### Reagents and antibodies

Trypsin-ethylene-diaminetetraacetic acid and DMEM medium were purchased from Gibco (Grand Island, NY, USA); Fetal bovine serum was from China Hangzhou Sijiqing Biological Technology Co.,Ltd.; 3-(4,5-dimethyl-2-thiazolyl)-2,5-diphenyl-2-H-tetrazolium bromide (MTT) and dimethylsulfoxide (DMSO) were provided by Sigma Chemical Co. (St. Louis, MO,USA); Caspase 3, E-cadherin, Vimentin and β-actin primary monoclonal antibody were purchased from Abcam Ltd. (Cambridgem MA, USA); Matrigel was from BD Biosciences (Los Angeles, CA, USA); Crystal violet was from Beijing Solarbio Science and Technology Co., Ltd.; Trizoland SuperScript™ III Reverse Transcriptase was from Invitrogen; RT Primers were synthesized by Invitrogen Biotechnology Co. Ltd.; PCR kit was from Arraystar INC. miRCURYTM Array Power Labeling kit and miRCURY™ Array were from Exiqon.

### Cell line and cell culture

HepG2, HepG2-luc and MHCC97H liver cancer cell lines were preserved in Beijing Institute of Hepatology. Cells were cultured in DMEM medium supplemented with 10% fetal bovine serum and maintained at 37 °C in a humidified incubator with 5% CO2.

### Animals and animal feeding

Four-week-old male balb/c nude mice (from Beijing Vital River Laboratory Animal Technology Co. Ltd) were raised and maintained in individually ventilated cages (IVC) under specific pathogen free sterile condition.

### MTT assay

Cells in the logarithmic growth phase were plated in 96-well plates in a seeding density of 2500–3000 cells per well and incubated in a 37 °C incubator with 5% CO2 overnight. After cells were treated with different concentration ESC for 24, 48, 72 h, the culture medium in each well was abandoned, incubating with 0.5 g/L MTT 100 μL for 4 h. Then each well was added with 150 μL DMSO and vibrated for 10 min, and absorbance of each well was detected with microplate reader (ELX800 type, BIO-TEX Instruments, INC, Winooski, VT, USA) at the 490 nm wave length. The inhibition rate (IR) was calculated as follows: IR (%) = (1 − OD_treatment_/OD_control_) × 100%. Half-maximal inhibitory concentration (IC_50_) was determined by logistic method.

### Transwell assay

The migration experiment was analyzed in 24-well transwell plates (Corning Incorporated). 2 × 10^4^ cells in 100 μL of DMEM medium with 1% BSA and different concentrations of ESC were added to the top chamber and 500 μL of 10% serum-containing DMEM was added in the bottom chamber. The cells were then incubated at 37 °C with 5% CO2 for 24 h. After incubation, the medium was removed, and non-invading cells were scrubbed by a wet cotton swab. The invading cells were washed by PBS for three times and fixed by 4% paraformaldehyde for 15 min. Fixed cells were washed three times by PBS and stained by 0.1% crystal violet in PBS for 10 min. Excess stain was washed by distilled water for three times. The migration cells were counted in five random fields in the same area for unbiased measurement using an inverted microscope.

### Western blot assay

Cells were seeded in 100 mm tissue culture dishes at the density of 2 × 10^6^ cells per dish and incubated for overnight. Cells were then treated with various agents as indicated in figure legends, then washed with ice-cold PBS and harvested in 400 μL of cell lysis buffer. The protein concentrations of lysates were determined using the bicinchonininc acid method. Cell lysates (40 μg protein per lane) were separated using 10% SDS-PAGE and transferred electrophoretically to polyvinylidenediluoride membrane. Membranes were blocked with tris-buffered saline/0.1% tween 20 containing 5% bovine serum albumin and then incubated overnight at 4 °C with primary antibodies (1:1000). Membranes were washed three times with TBST and incubated for 1 h at room temperature with the appropriate secondary antibody conjugated to goat anti-rabbit horseradish peroxidase (1:2000). Membranes were then washed and immunoreactive bands were developed with ECL and visualized by autoradiography. Protein loading was normalized using β-actin antibody. Gray-scale analysis of protein bands was performed using image software.

### Immunofluorescence analysis

Cells were seeded into 24-well plates and treated as described as figure legends. Cells were fixed with 4% formaldehyde for 30 min, washed with PBS, blocked with 5% BSA for 30 min at room temperature, and then stained with anti-human primary antibody (1:100) at 4 °C overnight. Cells were incubated with anti-rabbit-FITC secondary antibody (1:500) for 2 h at 4 °C, and then washed with PBS. Cells were then incubated for 10 min at room temperature with DAPI to stain nuclei, washed twice with PBS, and observed using an inverted fluorescence microscope (Olympus, Japan).

### Influence of ESC on Xenograft tumor models

3 × 10^6^HepG2-luc cells were inoculated in nude mice subcutaneously to establish xenograft tumors which must be transplanted into nude mice for 3 generations. The tumor tissues in the growth period were cut into about 1.5 mm^3^ and were inoculated in the right armpit skin and liver of nude mice under the condition of sterile. 3–6 days after transplantation, the mice were divided into groups according to the tumor growth observed by in vivo imaging system. Every group contained 6 mice. Then the mice were injected intraperitoneally with ESC indifferent dose according to the MTD (maximum tolerant dosage) experiment. The sizes of subcutaneous tumors were measured with caliper and in vivo imaging and orthotopic tumors were observed by in vivo imaging. At the endpoint of the experiment, nude mice were sacrificed with cervical dislocation. The tumor samples were collected according to the follow-up experiments. The tumor growth inhibition rates and relative tumor volumes (RTV) were calculated, RTV=Vt/V0 (Vt, the volumes of tumors of every calculation; V0, the volumes of tumors of initial administration).

### miRNA expression profiles of cancer cells treated with ESC

The experimental MHCC-97H cells were treated with the ESC (final concentration was 25 mg/mL) for 24 h. The control MHCC-97H cells were treated with vehicle agent for 24 h. Total RNA of cells was isolated using trizol and purified with RNeasy mini kit (QIAGEN) according to manufacturer’s instructions. RNA quality and quantity were measured by using nanodrop spectrophotometer (ND-1000, Nanodrop Technologies) and RNA integrity was determined by gel electrophoresis.

RNA labeling and array hybridization were according to Exiqon’s manual.1μLRNA in 2.0 μL of water was combined with 1.0 μL of CIP buffer and CIP (Exiqon). The mixture was incubated for 30 min at 37 °C. The reaction was terminated by incubation for 5 min at 95 °C. Then 3.0 μL of labeling buffer, 1.5 μL of fluorescent label (Hy3TM), 2.0 μL of DMSO, 2.0 μL of labeling enzyme were added into the mixture. The labeling reaction was incubated for 1 h at 16 °C. Terminated by incubation for 15 min at 65 °C. After stopping the labeling procedure, the Hy3™-labeled samples were hybridized on the miRCURYTM LNA Array (v.18.0) (Exiqon) according to array manual. The total 25 μL mixture from Hy3™-labeled samples with 25 μL hybridization buffer were first denatured for 2 min at 95 °C, incubated on ice for 2 min. Then hybridized to the microarray for 16–20 h at 56 °C in a Hybridization Systems (Hybridization System-Nimblegen Systems, Inc., Madison, WI, USA). Following hybridization, the slides were achieved, washed several times using Wash buffer kit (Exiqon). Then the slides were scanned using the Axon GenePix 4000B microarray scanner (Axon Instruments, Foster City, CA).

### Evaluation of the expression level of miRNAs and target genes

The expression level of miRNAs and target genes were measured and validated with real-time PCR. The methods were described as follows: 1.0 μg RNA, 1 μL0.5 μg/μLOligo (dT), 1.6 μL DNTPs Mix (2.5 mM) and RNAase free water 10.9 μL were made into annealing mixture.

The mixture was at 65 °C for 5 min and on ice for 2 min. After brief centrifugation, centrifugal pipe was added sequentially with RT mixture (4 μL 5X First-Strand Buffer, 1 μL 0.1 μM DTT, 0.3 μL RNase Inhibitor and 0.2 μL SuperScript III RT).

Then the mixture was incubated at 37 °C for 1 min, at 50 °C for 1 min, at 70 °C for 15 min, then the cDNAs were stored − 20 °C to be used.

PCR Realtime reaction system contained 5 μL 2 × Master Mix, 0.5μL10μM PCR forward primer, 0.5μL10μM PCR Reverse primer and 4 μL ddH_2_O. Primer sequences of validating genes were in Table 1.

**Table 1:** ᅟ

Validating genes	Primer sequences
has-miR-638	F:5’-ATCCAGTGCGTGTCGTG-3’
	R:5’-TGCTAGGGATCGCGGGCGGGTG-3’
has-miR-107	F:5’-ATACCGCTCGAGTGCCATGTGTCCACTGAAT-3’
	R:5’-ATACCGCTCGAGTTCCATGCCTCAACTCCTCT-3’
has-miR-106-5p	F:5’-GGGGGTAAAGTGCTGACAGT-3’
	R:5’-GTGCGTGTCGTGGAGTCG-3’
U6	F:5’-GCTTCGGCAGCACATATACTAAAAT-3’
	R:5’-CGCTTCACGAATTTGCGTGTCAT-3’
CDK2	F:5’-GTGGGCCCGGCAAGATTTTAG-3’
	R:5’-GCCGAAATCCGCTTGTTAGGG-3’
SOX2	F:5’-CACATGAAGGAGCACCCGGATTAT-3’
	R:5’-GTTCATGTGCGCGTAACTGTCCAT-3’
STAT3	F:5’-TGGAAATAATGGTGAAGGTGC-3’
	R:5’-ATCTGGGGTTTGGCTGTGT-3’
Bcl2	F:5’-AGTGGGATGCGGGAGATGTG-3’
	R:5’-GGGATGCGGCTGGATGGG-3’
Mcl1	F:5’-TAAGGACAAAACGGGACTGG-3’
	R:5’-ACCAGCTCCTACTCCAGCAA-3’
CACNA2D1	F:5’-GACTGACCAACACCACTCTTCAC-3’
	R:5’-CT ATCGTACCTCAGCTCCTTCC-3’
SALL4	F:5’-CCAAAGGCAACTTAAAGGTTCAC-3’
	R:5’-CCGTGAAGACCAATGAGATCTCC-3’

After the reaction was mixed and centrifuged with 5000 rpm, 8 μl mixed solution was added into 384PCR plates, and then 2 μL cDNA was added correspondingly. The reaction system was centrifuged briefly to mix. Three hundred eighty-four plates were placed on the PCR instrument (BIO-RAD) and all indicators were carried out according to the following procedures: 95 °C, 10 min; 40 circles of PCR(95 °C, 10s;60 °C,60 s). To establish the melting curve of PCR products, after amplified reaction was over, procedures were accorded as follows: °C,10s; 60 °C,60s; 95 °C,15 s and temperature was increased slowly from 60 °C to 95 °C(0.05 °C/s). The relative increase in reporter fluorescent dye emission was monitored. The level mRNA, relative to actin, was calculated using the formula: Relative mRNA expression = 2^ [ct (validating genes_control_) – ct (validating genes_ESC_) + ct (U6_ESC_) – ct (U6_control_)], where ct is defined as the number of the cycle in which emission exceeds an arbitrarily defined threshold.

### Statistical analysis

All data are the means of three determinations and data were analyzed using the SPSS Package for Windows (Version 16). Statistical analysis of the data was performed with ANOVA. Differences with *P* < 0.05 were considered statistically significant.

## Results

### Growth inhibition of ESC in HepG2 cells and MHCC97H cells

**Fig. 1:**
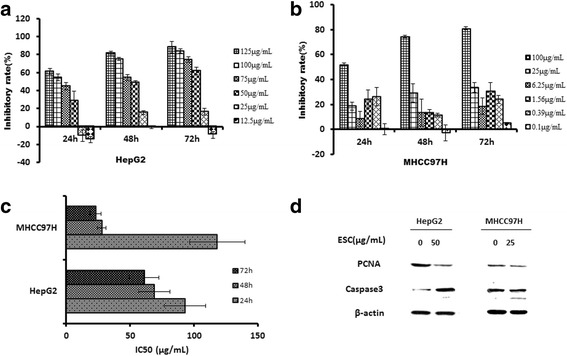
Growth inhibition of ESC in HepG2 cells and MHCC97H cells. **a** Inhibition rates of ESC in HepG2 cells at 24, 48,72 h. **b** Inhibition rates of ESC in MHCC97H cells at 24, 48,72 h. **c** IC50 values of ESC in HepG2 and MHCC97H cells at 24, 48,72 h. **d** Influence of ESC on PCNA and caspases 3 expression in HepG2 cells and MHCC97H cells with western blot method

The inhibition rates of 25–125 μg/mL ESC at 24 h in HepG2 cells were respectively − 10.28%, 28.37%, 44.82%, 54.63%, 61.33%; the inhibition rates of 25–125 μg/mL ESC at 48 h HepG2 cells were respectively 15.62%,48.59%,54.32%,74.97%,81.52%; the inhibition rates of 25–125 μg/mL ESC at 72 h HepG2 cells were respectively 16.16%,61.89%,74.32%,84.09%,88.23% (Fig. 1a). IC50 values at 24,48,72 h were respectively 92.95, 68.96, 61.06 μg/mL (Fig. 1c). These results suggested 25–125 μg/mL ESC had a significant inhibitory effect on HepG2 cells and this effect showed a certain dose and time dependence. 50 μg/mL ESC can significantly inhibit of PCNA and increase Caspase3 protein expression at 24 h in HepG2 cells (Fig. 1d). The inhibition rates of 0.39–125 μg/mL ESC at 24 h in MHCC97H cells were respectively 26.27%, 24.37%, 8.38%, 18.65%,52.03%; the inhibition rates of 0.39–125 μg/mL ESC at 48 h in MHCC97H cells were respectively 11.39%,13.63%,13.50%,29.06%,74.30%; the inhibition rates of 25–125 μg/mL ESC at 72 h in MHCC97H cells were respectively 24.24%,30.56%,18.60%,33.67%,81.03% (Fig. 1b). IC50 values at 24,48,72 h were respectively 118.23, 27.79, 23.08 μg/mL (Fig. 1c). These results suggested 0.39–125 μg/mL ESC had a significant inhibitory effect on MHCC97H cells and this effect showed certain dose dependence. 25 μg/mL ESC can also slightly inhibit of PCNA and increase Caspase3 protein expression at 24 h in MHCC97H cells (Fig. 1d).

### EMT inhibition of ESC in HepG2 cells and MHCC97H cells

**Fig. 2:**
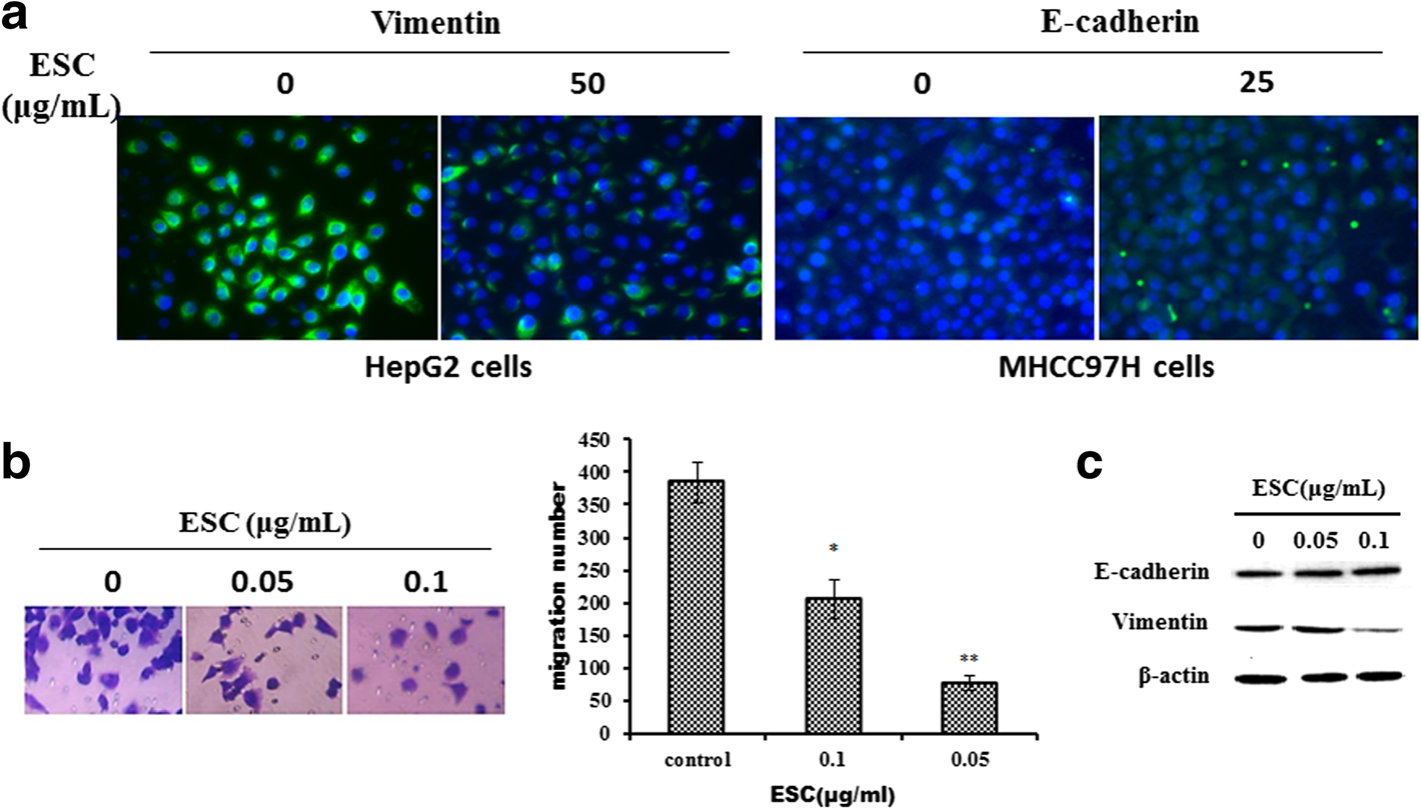
EMT inhibition of ESC in HepG2 cells and MHCC97H cells. **a** Influence of ESC on Vimentin and E-cadherin expression in HepG2 cells and MHCC97H cells with immunofluorescence assay at 24 h. **b** Influence of ESC on MHCC97H cells migration with transwell assay at 24 h. **c** Influence of ESC on Vimentin and E-cadherin expression in MHCC97H cells with western blot method at 24 h. *p < 0.05, **p < 0.01*vs* control

Immunofluorescence results showed that 50 μg/mL ESC can obviously reduce the expression of vimentin protein inHepG2 and 25 μg/mLESC can significantly upregulate E-cadherin protein expression in MHCC97H cells (Fig. 2a). In MHCC97H, less than inhibitory concentration of 0.05 μg/mL ESC can inhibit obviously cell migration using transwell assay and the expression of vimentin with western blot method (Fig. 2b and c). These results suggested that ESC can inhibit EMT in HepG2 cells and MHCC97H cells.

### Growth and metastasis inhibition of ESC on xenograft models of HepG2 cells

**Fig. 3:**
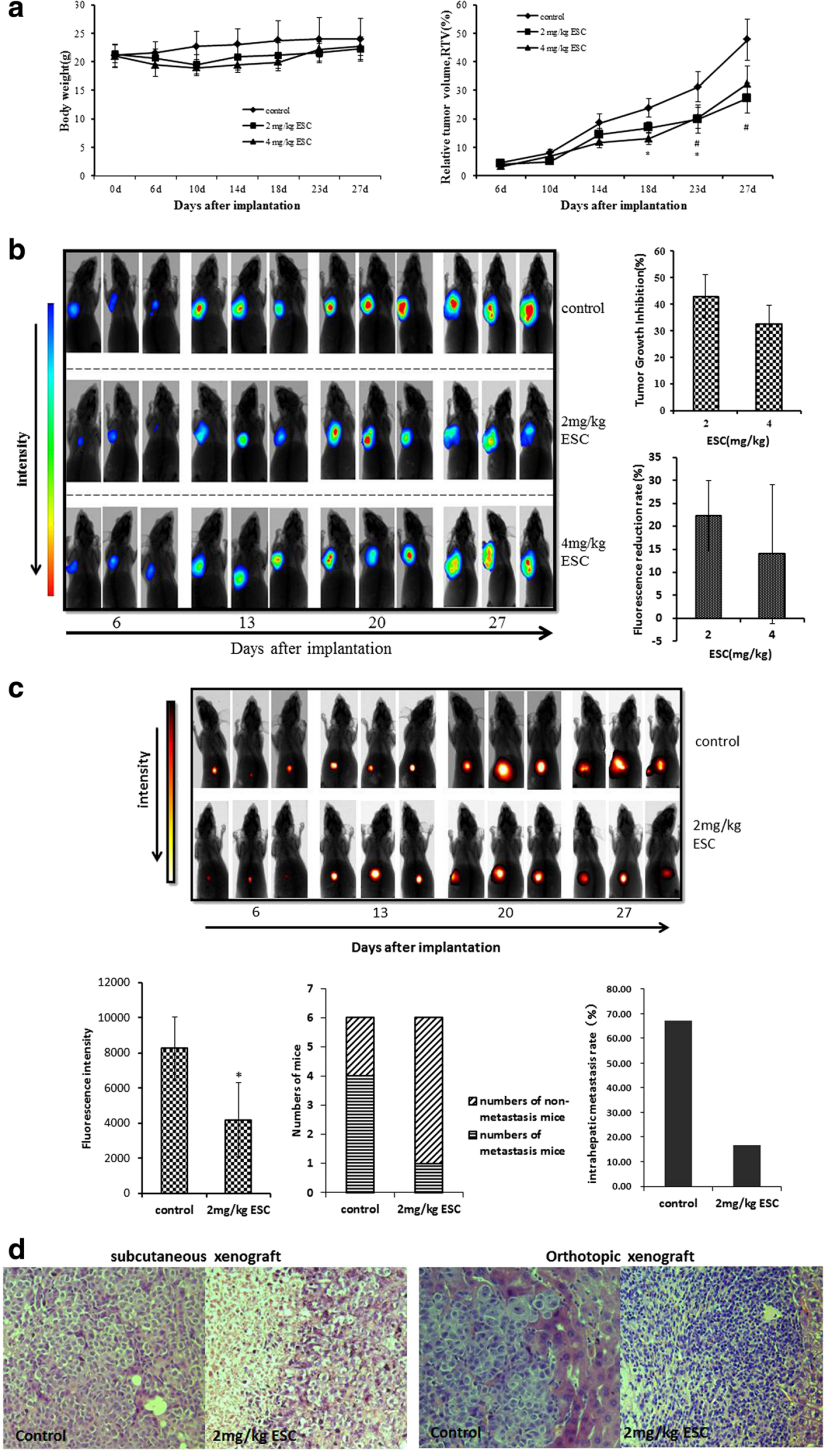
Growth and metastasis inhibition of ESC on xenograft tumor models mice of HepG2 cells. **a** Changes of body weight and RTV of subcutaneous xenograft model mice of HepG2cells after treated with ESC. **b** Influence of ESC on subcutaneous xenograft tumor models mice of HepG2 cells by in vivo imaging. **c** Influence of ESC on orthotopic xenograft tumor models mice of HepG2 cells by in vivo imaging. **d** Hemetoxylin and Eosin Stains of xenograft tumor tissues (× 100)

Nude mice bearing tumors could be tolerated 2,4 mg/kg doses of ESC, and the weight loss was always less than 20% during the whole experiment. 2,4 mg/kg ESC could significantly inhibit the relative tumor volume of subcutaneous xenograft hepatocellular carcinoma (Fig. 3a). In vivo imaging showed that 2,4 mg/kg ESC significantly inhibited fluorescence density value of tumor interior. Tumor growth inhibition rates of 2,4 mg/kg ESC were respectively 43.01% and 32.49%. Fluorescence reduction rates of 2,4 mg/kg ESC were respectively 22.29% and 14.03%(Fig. 3b). 2 mg/kg ESC could also significantly inhibit fluorescence density value of tumor interior in orthotopic xenograft nude mice and inhibited intrahepatic metastasis rate. Intrahepatic metastasis rates of control and 2 mg/kg ESC groups were respectively 67% and 16.67%. Fluorescence reduction rate of 2 mg/kg ESC was 49.30% (Fig. 3c). Pathological tissue showed severe necrosis in tumor tissues of subcutaneous xenograft nude mice and infiltration by inflammatory cells in orthotopic tumor tissues after treatment with ESC (Fig. 3d). These results suggested that ESC could inhibit the growth and metastasis of hepatocellular carcinoma.

### miRNA expression profiles of HCC cells treated with ESC

**Fig. 4:**
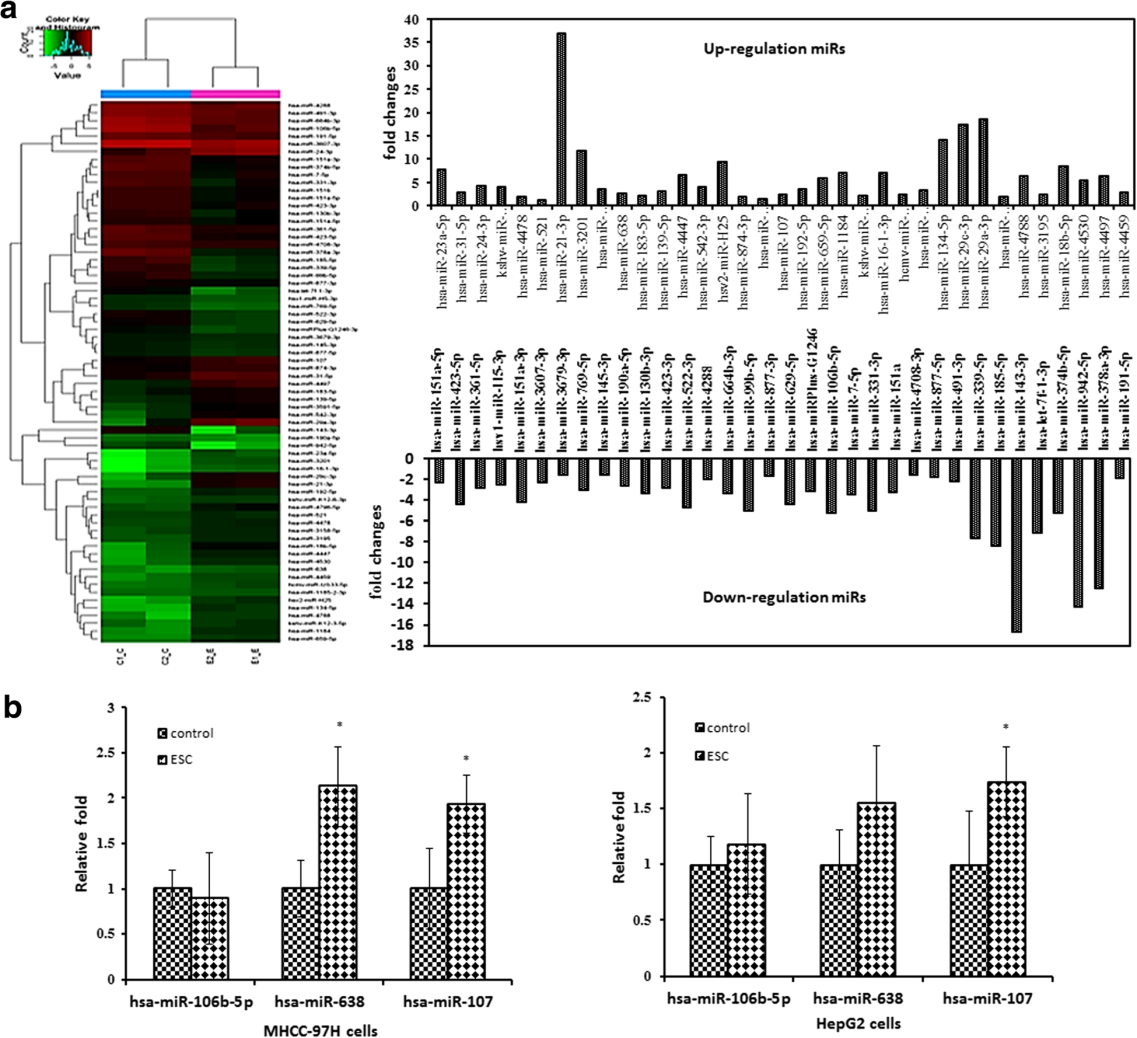
miRNA expression profiles of HCC cells treated with ESC. **a** Profile of miRNAs of MHCC97H cells treated with ESC. **b** Part differential miRNA expressions were verified with realtime PCR in MHCC97H and HepG2 cells. *p < 0.05, **p < 0.01*vs* control

RNAs ofMHCC97H cells were extracted to screen differential miRNAs after being treated with 25 μg/mL ESC. 35 up-regulated and 34 down-regulated miRNAs were detected in total of 429 miRNAs in accordance with the standard (fluorescence value change> 1.5 times and have significant difference between ESC and control group) (Fig. 4a). Among of them, 12miRNAs (hsa-miR-139-5p, hsa-miR-638, hsa-miR-107, hsa-miR-331-3p, hsa-miR-21-3p, hsa-miR-134-5p, hsa-miR-16-1-3p, hsa-miR-339-5p, hsa-miR-106b-5p, hsa-miR-423-3p, hsa-miR-491-3p, hsa-miR-24-3p) were related to cancers. According to the professional knowledge and literature (Table 2), 3 differential miRNAs (hsa-miR-107, hsa-miR-638, hsa-miR-106b-5p) that related to cancers were singled out for real-time PCR validation. Real-time PCR results showed that hsa-miR-107, hsa-miR-638 expressions were obviously different in MHCC97H cells and the difference of hsa-miR-107 in the HepG2 cells was obviously observed (Fig. 4b).

**Table 2:** Part differential miRNAs involving with cancers

miRNAs	Fold change	*P*-value	miRNA related to cancers	References
has-miR-638	2.97	0.02	Non-small cell lung cancer	[24–26]
			Melanoma	[27]
			Leukemia	[28]
			Hepatocellular cancer	[29]
			Colorectal carcinoma	[29–32, 15]
			Breast cancer	[33]
			Gastric cancer	[34]
			Cervical cancer	[35]
has-miR-107	2.56	0.04	Gastric adenocarcinoma	[36, 37]
			Glioma	[38, 39]
			Non-small cell lung cancer	[40]
			Cervical cancer	[14]
			Leukemia	[41]
			Colorectal cancer	[42]
			Neck squamous cell carcinoma	[15]
has-miR-106-5p	0.19	0.03	Pancreatic cancer	[43]
			Renal carcinoma	[44]
			Glioma	[45]
			Colorectal cancer	[13]
			Breast cancer	[46]

### Evaluation of the expression level of target genes

**Fig. 5:**
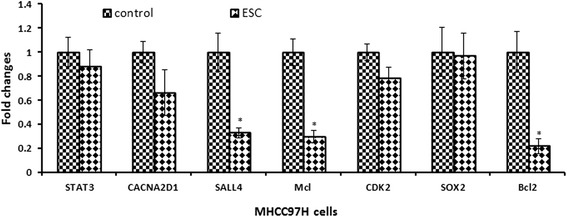
Evaluation of the expression level of target genes. Target genes STAT3, CACNA2D1, SALL4, MCL1, CDK2, SOX2 and Bcl2 of miRNAs were validated with real-time PCR in MHCC-97H cells. ESC could significantly downregulate SALL4, McL-1 and Bcl-2 genes that has-miR-107 targeted in MHCC-97H cells. *p < 0.05, **p < 0.01 vs control

According to the bioinformatics prediction and references (Table 3), we chose target genes STAT3, CACNA2D1, SALL4, MCL1, CDK2, SOX2 and Bcl2 for real-time PCR validation. The results showed that ESC could significantly down regulate SALL4, McL-1 and Bcl-2 genes in HCC cells (Fig. 5).

**Table 3:** Target genes of different miRNAs

iniRNAs	Target genes	Databases of bioinformatics prediction	References
has-miR-638	CDK2		[24–26]
	SOX2		[27]
has-miR-106-5p	STAT3	Miranda; mirbase; targetscan	[36, 37, 47]
has-miR-107	MCL1		[38, 40]
	CACNA2D1	miranda; mirbase; targetscan	[40]
	SALL4	Miranda; mirbase; targetscan	[14]
	Bcl2		[41]

## Discussion

In this study, ESC showed promising inhibitory effect on growth of HepG2 and MHCC97H cells in dose and time dependent style. MHCC97H cells were more sensitive to ESC. ESC also obviously inhibited apoptotic protein caspase 3 and proliferative protein PCNA in these two cell lines. ESC inhibited EMT protein vimentin in HepG2 cells and upregulated E-cadherin in MHCC97H cells**.** ESC of low concentration might regress the migration of MHCC97H cells and inhibit EMT protein vimentin expression. Interestingly, the lowest dose (0.05 μg/mL) of ESC inhibited the cell migration more effectively than the highest dose (0.1 μg/mL). However, 0.1 μg/mL of ESC was more effective to increase the expression of E-cadherin and decrease that of vimentin. The contradiction result may be related to the complexity of the ESC components. No matter how, these results suggested that ESC could inhibit proliferation and EMT in HCC cells in vitro*.* In vivo results showed that ESC significantly inhibited tumor growth in either subcutaneous or orthotopic xenograft models mice of HepG2. ESC also had certain inhibitory effect on intrahepatic metastasis. All these results suggested that ESC was an important potential inhibitor of hepatocellular carcinoma.

Plenty of studies have shown that miRNAs play fundamental roles in many pathological processes. Meanwhile accumulating evidences in cancer diagnostics and therapeutics indicate that miRNAs involve in HCC progression, which may serve as either sensitive biomarkers for detecting carcinogenesis as well as monitoring therapies of HCC or tumor suppressors or oncogenes [10–12]. As we all know, Traditional Chinese Medicine (TCM) has the characteristics of multiple effective targets on the diseases and we reasonably hypothesized that ESC might exert anti-tumor role by regulating microRNAs. Here, the anti-cancer mechanisms of ESC targeting miRNAs have been extensively explored, and we chose miRNA array to detect the miRNA profile of MHCC97H cells treated with ESC. 69 differential miRNAs in total of 429 ones were obtained. According to the references, we selected hsa-miR-107, hsa-miR-638, hsa-miR-106b-5p to be verified with real-time PCR in MHCC97H and HepG2 cells. Expressions of hsa-miR-107, hsa-miR-638 in HCC cells treated by ESC were significantly increased. Hsa-miR-107, which functionally overlaps with miR-15, miR-16, and miR-195 due to a common 5′ sequence critical for target specificity [13]. There were opposite arguments in roles of hsa-miR-107 on cancers. Hsa-miR-107 activated ATR/Chk1 pathway, suppressed cervical cancer invasion and inhibited the tumorigenicity of head and neck squamous cell carcinoma [14, 15]. hsa-miR-107 is also confirmed to be involved in the progression of HCC [12, 16]. In this study, we found ESC could upregulate the expression hsa-miR-107 in both MHCC97H and HepG2 cells. Increased expression of hsa-miR-638 was only observed in MHCC97H cells treated by ESC, while the expression of hsa-miR-106b-5p did not change in any of these two cancer cell lines. These results confirmed that ESC could inhibit hepatocellular carcinoma by regulating some miRNAs.

Correspondingly, the target genes of hsa-miR-107, hsa-miR-638, hsa-miR-106b-5p were measured with PCR assay. Interestingly, only target genes of hsa-miR-107 were changed greatly. From the references and databases of bioinformatics, we have known that MCL1, CACNA2D1, SALL4 and Bcl2 were target genes of hsa-miR-107. Discovered as crucial modulators of apoptosis, anti-apoptotic Bcl-2 protein family emerged more recently as important modulators of other essential cancer processes, including cell cycle, autophagy or cell metabolism. Most cancer cell models overexpress one or more of the three major proteins: BCL-2 (B-cell lymphoma 2), BCL-xL (B-cell lymphoma-extra-large) and MCL1 (myeloid cell leukemia1). MCL1 and BCL-2 could enhance cell survival by inhibiting apoptosis [17, 18]. CACNA2D1(calcium voltage-gated channel auxiliary subunit alpha2delta 1) genes was critical for HCC TIC (tumor initiating cell) stemness and was predictive of poor prognosis for HCC patients [19, 20]. SALL4 (spalt like transcription factor 4), a member of a family of zinc finger transcription factors, was a marker for a progenitor subclass of HCC with an aggressive phenotype and a regulator of embryogenesis, organogenesis, pluripotency**.** Upregulation of SALL4 was also associated with poor prognosis in HCC [21–23]. The real-time PCR validation showed that ESC downregulated the MCL1, SALL4 and BCL2 gene expression significantly, but did not influence the expression of CACNA2D1. These results illustrated that ESC might regulate target genes of miRNAs, but what regulating style, direct or indirect, was still needed to be further explored in future.

## Conclusion

ESC regressed growth and metastasis of human hepatocellular carcinoma. ESC obviously upregulated hsa-miR-107 and hsa-miR-638 expression, but only target genes of hsa-miR-107, MCL1, SALL4 and Bcl2, were changed greatly. These findings suggested that regulating microRNAs expression and their corresponding target genes might the one of important molecular mechanisms of ESC treatment with HCC.

## Abbreviations

BCL2: B-cell lymphoma 2
CACNA2D1: Calcium voltage-gated channel auxiliary subunit alpha2 delta 1
CDK2: Cyclin-dependent kinase 2
DMSO: Dimethyl sulfoxide
EMT: Epithelial mesenchymal transition
ESC: Extract of Stellerachamaejasme
HCC: Hepatocellular carcinoma
IC50: Half-maximal inhibitory concentration
IR: Inhibition rate
IVC: Individually ventilated cage
MCL1: Myeloid cell leukemia-1
MTT: 3-(4,5- dimethyl- 2-thiazolyl)-2,5-diphenyl-2-H-tetrazolium bromide
PCR: Polymeras chain reaction
RT: Reverse transcription
RTV: Relative tumor volume
SALL1: Spalt like transcription factor 4
SOX2: Sex determining region Y box 2
STAT: Signal transducers and activators of transcription
TGF: Transforming growth factor
TIC: Tumor initiating cell
